# Causes and correlates of 30 day and 180 day readmission following discharge from a Medicine for the Elderly Rehabilitation unit

**DOI:** 10.1186/s12877-018-0883-3

**Published:** 2018-08-28

**Authors:** Lloyd D. Hughes, Miles D. Witham

**Affiliations:** 1 GP Registrar, Primary Care Directorate, NHS Education for Scotland, Edinburgh, UK; 2 Ageing and Health, University of Dundee, Ninewells Hospital, Dundee, UK

## Abstract

**Background:**

Recently hospitalized patients experience a period of generalized risk of adverse health events. This study examined reasons for, and predictors of, readmission to acute care facilities within 30 and 180 days of discharge from an inpatient rehabilitation unit for older people.

**Methods:**

Routinely collected, linked clinical data on admissions to a single inpatient rehabilitation facility over a 13-year period were analysed. Data were available regarding demographics, comorbid disease, admission and discharge Barthel scores, length of hospital stay, and number of medications on discharge. Discharge diagnoses for the index admission and readmissions were available from hospital episode statistics. Univariate and multivariate Cox regression analyses were performed to identify baseline factors that predicted 30 and 180-day readmission.

**Results:**

A total of 3984 patients were included in the analysis. The cohort had a mean age of 84.1 years (SD 7.4), and 39.7% were male. Overall, 5.6% (*n* = 222) and 23.2% (*n* = 926) of the patients were readmitted within 30 days and 180 days of discharge respectively. For patients readmitted to hospital, 26.6% and 21.1% of patients were readmitted with the same condition as their initial admission at 30 days and 180 respectively. For patients readmitted within 30 days, 13.5% (*n* = 30) were readmitted with the same condition with the most common diagnoses associated with readmission being chest infection, falls/immobility and stroke. For patients readmitted within 180 days, 12.4% (*n* = 115) of patients were readmitted with the same condition as the index condition with the most common diagnoses associated with readmission being falls/immobility, cancer and chest infections. In multivariable Cox regression analyses, older age, male sex, length of stay and heart failure predicted 30 or 180-day readmission. In addition, discharge from hospital to patients own home predicted 30-day readmission, whereas diagnoses of cancer, previous myocardial infarction or chronic obstructive pulmonary disease predicted 180-day readmission.

**Conclusion:**

Most readmissions of older people after discharge from inpatient rehabilitation occurred for different reasons to the original hospital admission. Patterns of predictors for early and late readmission differed, suggesting the need for different mitigation strategies.

## Background

Readmission after discharge from hospital is common and has a considerable cost [1]. In the USA nearly one fifth of Medicare patients discharged from a hospital (approximately 2.6 million seniors), have an acute medical problem within 30 days that requires a further admission for treatment [2]. Furthermore, there is evidence that patients that are readmitted have a longer length of stay than for first admissions and a higher risk of complications [3].

The days and weeks after hospital discharge are a time of high risk not only for recurrence of the index medical condition, but for a wide range of other health and social care problems. Consequently, a majority of readmissions in older people are due to a diagnosis other than the index admission diagnosis [2]. This observation has led to the concept of a ‘post-hospitalisation syndrome’, described as an acquired transient period of vulnerability [4]. This syndrome may extend beyond the 30 days commonly used as the benchmark for readmission rates, perhaps as long as 6 months after the index admission [5].

Because of the risks and costs associated with readmission, there is considerable interest in identifying which patients are at risk of readmission, with a view to intervening to reduce readmission rates. The use of readmission rates as a quality standard in healthcare gives further impetus to these efforts. There has been some work developing predictive models to assist in the reduction in readmission rates, with varying degrees of success [6–9]. The majority of studies in this area to date have however excluded patients discharged to nursing homes and have focused on patient discharges from acute receiving hospitals. Indeed, predictive algorithms for readmission [1, 5, 6] have not specifically studied older patients, who may have differing reasons for readmission compared to younger patients.

There are also limited data on readmission rates for patients who have experienced a period of in-patient rehabilitation after a period of prolonged illness, with evidence to date from American studies. These patients typically remain in hospital for a number of weeks, and thus subsequent readmissions may be less likely to be related to hasty or incomplete discharge planning, allowing the impact of post-hospitalisation syndrome rather than incomplete discharge planning and community support to be dissected out.

Ottenbacher et al. reviewed centrally held data from 1365 post-acute inpatient rehabilitation facilities (*n* = 736,536), reported 30-day readmission rates of between 5.8 and 18.8% for different sub-groups of patients [10]. 50% of readmissions were within 11 days. The same research group have published further work focusing upon patients with ‘debility’, and reported higher rates of hospital readmission of 19% at 30 days and 34% at 90 days [11]. There are considerable differences between the manner in which rehabilitation is provided in the USA and in Europe (in relation to providers of care, differing financial incentives, type of rehabilitation facilities where care is provided) meaning that these findings may not be directly comparable.

This study therefore aimed to use routinely collected healthcare data to establish a) the reasons for readmission to acute care facilities in a cohort of older people discharged from inpatient rehabilitation after an acute illness, b) whether the reasons for readmission varied by the reason for the index admission, and c) what the predictors for 30 and 180 day readmission were in this cohort.

## Methods

### Service characteristics

The Dundee Medicine for the Elderly rehabilitation service offers inpatient rehabilitation to patients located within Dundee (Scotland, United Kingdom) unitary authority (population 150,000). Patients over the age of 65 years, are accepted to the unit following an admission at acute receiving hospitals for acute medical or surgical illness from a variety of specialties including general medicine, general surgery, orthopedics, stroke medicine and neurosurgery. Patients are also accepted from sub-acute Medicine for the Elderly wards. Patients were selected following review by a consultant geriatrician; patients selected were those felt to have potential to achieve independence in domains of self-care who were medically stable after their acute admission. Patients who had limited to no expectation of functional improvement within a reasonable period of time or those felt unlikely to survive to discharge were not selected for transfer to the rehabilitation unit.

Inpatient rehabilitation is carried out on dedicated rehabilitation wards by a multidisciplinary team, including physiotherapists, occupational therapists, dieticians, social workers and speech and language therapists. This process is over-seen by a consultant geriatrician, with patient progress meetings at weekly intervals to discuss progress and any issues that may affect discharge success. The model of care on the rehabilitation unit remained unchanged throughout the analysis period.

### Data sources

This analysis was conducted using linked, routinely collected clinical data in Tayside, Scotland. Anonymised data are held by the University of Dundee Health Informatics Centre (HIC) in an access-controlled Safe Haven environment. Analysis complied with HIC Standard Operating Procedures approved by the NHS East of Scotland Research Ethics Service and the NHS Tayside Caldicott Guardian. Separate ethics review for this project was therefore not required.

### Data collected

Data used in this analysis were prospectively collected on all admissions to the Dundee Medicine for the Elderly rehabilitation unit between 1 January 1999 and 31 December 2011. Data were collected as part of routine clinical care and reviewed by the team caring for the patient during inpatient rehabilitation. The cohort was followed up until the end of May 2012. Mortality data were obtained using death certification information derived from Scottish Register Officer. This cohort has been described in detail previously [12–14].

Variables included age, sex, Scottish Index of Multiple Deprivation Quintile [15], discharge destination (home versus other options, which comprised long-stay hospital beds or care home), comorbid disease, admission and discharge 20-point Barthel scores, length of rehabilitation hospital stay, and number of medications on discharge. Comorbid disease diagnoses were obtained in two different ways. A diagnosis of chronic kidney disease was coded based upon estimated glomerular filtration rate (eGFR) taken from linked clinical data using the MDRD equation [16]. Other diagnoses were obtained using International Classification of Diseases (ICD) 10 discharge diagnosis codes from hospital admissions prior to the index acute admission [17]. These included a diagnosis of previous myocardial infarction, stroke, congestive cardiac failure, and chronic obstructive pulmonary disease (COPD). The presence of diabetes mellitus was ascertained from the Scottish Care Information – Diabetes Collaborative (SCI-DC) database.

In addition, information on dynamic changes in C-reactive protein (CRP) was obtained as a measure of biological resilience [18], including maximum-recorded value during admission and time taken for elevated levels to halve in value.

### Classification of admission and readmission diagnoses

The main diagnostic reason (recorded as ICD-10 codes) for admission to acute hospital prior to the rehabilitation referral for all patients was obtained from HIC datasets, alongside the main first readmission diagnosis to acute hospital for patients who were readmitted within 30 or 180 days. Only the first readmission was considered in this analysis.

These ICD codes were recorded, and collated into broader categories. For example, all cancer diagnoses were collated into ‘Cancer Diagnoses’ and different forms of dementia were collated into ‘Dementia States’. In the 30-day and 180-day readmission groups, the 10 most common reasons for admission to hospital were established after reviewing collated diagnoses lists. The diagnoses for readmission were then charted by initial admission diagnosis for each of the two readmission groups in order to establish any relationships between initial admission and readmission diagnoses.

### Data analysis

Statistical analyses were carried out in SPSS v22.0 (IBM, New York USA), and a two-sided *p* value of < 0.05 was taken as significant for all analyses. Individuals were excluded from the analysis if they died during their inpatient admission, or did not have a discharge Barthel score. The number of days between patients discharge from rehabilitation hospital discharge to next acute hospital admission was calculated, with readmission to acute hospital within 30 days and 180 days analysed separately. Cox regression analysis was used to examine the association between baseline factors and acute hospital readmission with dates censored at 30 days and 180 days after discharge (or at death if this was earlier). Analyses were adjusted for age, sex, and comorbid disease; variables with a *p*-value < 0.3 on univariate analysis were also entered into the adjusted model.

## Results

Of the 4449 patients in the complete medicine for the elderly rehabilitation dataset, 409 died during admission and were excluded from analysis, with a further 65 excluded due to the absence of a discharge Barthel score. A total of 3984 patients were included in the analysis.

### Baseline characteristics

The characteristics of the overall patients group, patients readmitted to acute hospital care within 30 days and 180 days of discharge from the rehabilitation hospital are given in Table 1. Twenty-nine patients died within 30 days of discharge without being readmitted, and 325 died within 180 days of discharge without being readmitted. Patients readmitted to acute hospital facilities within 30 or 180 days were more likely to have a diagnosis of cancer, chronic obstructive pulmonary disease, congestive cardiac failure, previous myocardial infarction, a higher number of general hospitalizations over the period of data-collection (1999–2012) and a higher admission Barthel score.

**Table 1:** Characteristics of overall cohort, patients readmitted within 30 days and patients readmitted within 180 days

	Whole cohort *n* = 3984	Readmitted by 30 days *n* = 222	Not readmitted and alive at 30 days (*n* = 3733)	Readmitted by 180 days *N* = 926	Not readmitted and alive at 180 days (*n* = 2733)	Died before readmission and before 180 days (*n* = 325)
Mean age (years) (SD)	84.1 (7.4)	82.7(7.3)*	84.2 (7.4)	83.6 (7.3)**	84.4 (7.4)	82.8 (7.5)
Male sex (%)	1582 (39.7)	112 (50.5)*	1459 (39.1)	419 (45.2)**	1017 (37.2)	146 (44.9)
Discharged to own home (%)	2982 (74.8)	161 (72.5)	2810 (75.3)	757 (81.7)**	2072 (75.8)	153 (47.1)
SIMD Quintiles (%)						
1 (most deprived)	1167 (29.3)	65 (29.3)	1090 (29.2)	277 (30.0)	784 (28.7)	106 (32.6)
2	572 (14.4)	31 (14.0)	536 (14.4)	127 (13.7)	388 (14.2)	57 (17.5)
3	490 (12.3)	27 (12.2)	459 (12.3)	109 (11.8)	333 (12.2)	48 (14.8)
4	1073 (26.9)	66 (29.7)	1002 (26.8)	241 (26.0)	758 (27.7)	74 (22.8)
5 (least deprived)	596 (15)	28 (12.6)	565 (15.1)	147 (15.9)	412 (15.1)	37 (11.4)
Missing Value	86 (2.2)	5 (2.3)	81 (2.2)	25 (2.7)	58 (2.1)	3 (0.9)
Mean Admission Barthel Score (SD)	10.4 (3.8)	11.1 (3.8)*	10.4 (3.8)	10.4 (3.6)	10.5 (3.8)	9.4 (4.3)
Mean Discharge Barthel Score (SD)	14.4 (4.6)	14.7 (4.4)	14.4 (4.6)	14.5 (4.3)	14.7 (4.5)	10.6 (6.0)
Median Length of Stay (days) (IQR)	33 (18–62)	28 (16–52)*	34 (18–62)	33 (20 – 56)	34 (18–64)	29 (18–55)
Median Number of Medications on Discharge (IQR)	2 (0–5)	2 (0–5)	2 (0–5)	3 (0–6)	3 (0–5)	0 (0–3)
Previous Myocardial Infarction (%)	683 (17.1)	52 (23.4)*	628 (16.8)	188 (20.3)**	435 (15.9)	60 (18.5)
Previous Stroke (%)	286 (7.2)	16 (7.2)*	266 (7.1)	67 (7.2)	189 (6.9)	30 (9.2)
Congestive Heart Failure (%)	342 (8.6)	33 (14.9)*	304 (8.1)	109 (11.8)**	183 (6.7)	50 (15.4)
eGFR < 60 ml/min/1.73m^2^ (%)	2019 (50.7)	109 (49.1)	1891 (50.7)	482 (52.1)	1373 (50.2)	164 (50.5)
eGFR 30–59	1090 (27.4)	57 (25.7)	1023 (27.4)	251 (27.1)	762 (27.9)	77 (23.7)
eGFR 15–29	709 (17.8)	40 (18.0)	664 (17.8)	170 (18.4)	473 (17.3)	66 (20.3)
eGFR < 15	220 (5.5)	12 (5.4)	204 (5.5)	61 (6.6)	138 (5.0)	21 (6.5)
COPD (%)	553 (13.9)	46 (20.7)*	502 (13.4)	157 (17.0)**	338 (12.4)	58 (17.8)
Previous Diagnosis of Cancer (%)	467 (11.7)	34 (15.3)	428 (11.5)	125 (13.5)**	262 (9.6)	80 (24.6)
Diabetes Mellitus (%)	709 (17.8)	50 (22.5)	654 (17.5)	168 (18.1)	501 (18.3)	40 (12.3)

*SIMD* Scottish Index Multiple Deprivation Quintiles, *SD* standard deviation, *IQR* inter-quartile range, *eGFR* estimated glomerular filtration rate, *COPD* Chronic obstructive pulmonary disease

*p < 0.05 vs group not readmitted at 30 days

**p < 0.05 vs group not readmitted at 180 days

#### Readmission to acute hospital diagnoses

For patients readmitted within 30 days, 27% (*n* = 59/222) of patients were readmitted with the same condition as their initial admission. For patients readmitted within 180 days, 21% (*n* = 196/926) of patients were readmitted with the same condition as their initial admission. The most common reasons for readmission for patients readmitted within 30 days were chest infection (*n* = 20), stroke (*n* = 14) and falls/immobility (*n* = 13). The most common reasons for readmission for patients readmitted within 180 days were admission secondary to falls/immobility (*n* = 99), chest infection (*n* = 55) or secondary to cancer (*n* = 51).

**Fig. 1:**
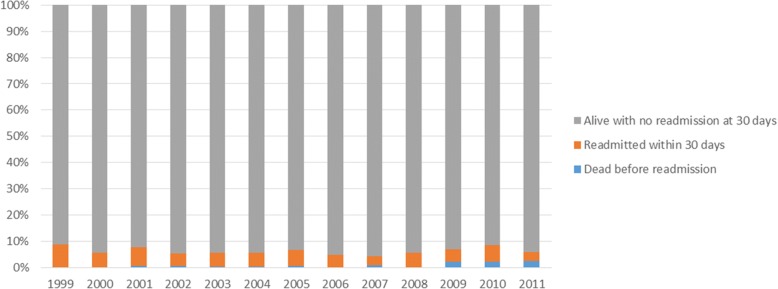
Death of Readmission within 30 days by year of index admission

**Fig. 2:**
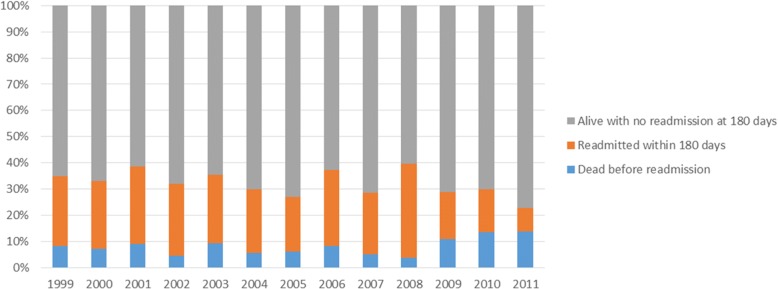
Death of Readmission within 180 days by year of index admission

**Fig. 3:**
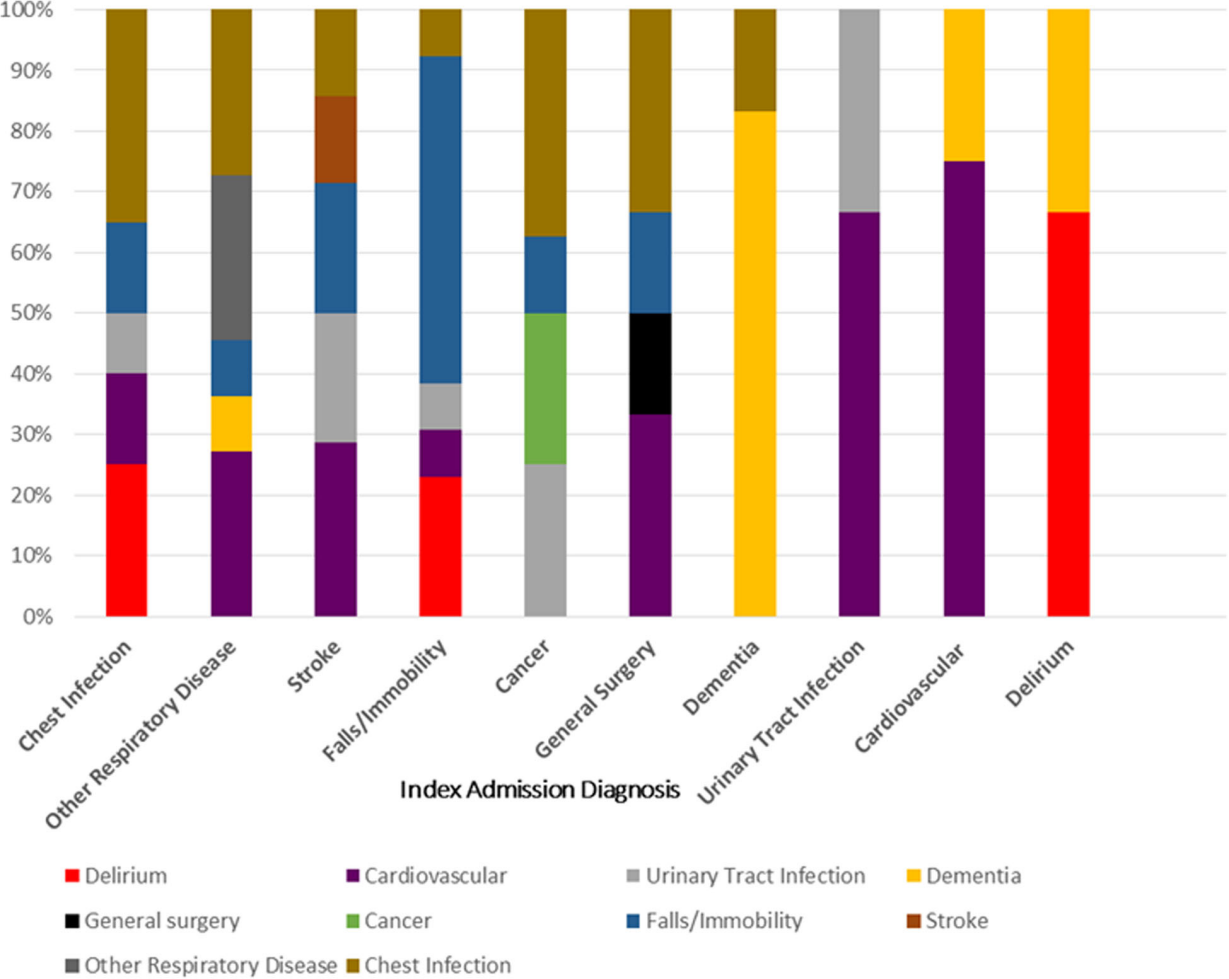
Diagnoses leading to readmission compared to first admission diagnoses (30 day readmissions)

**Fig. 4:**
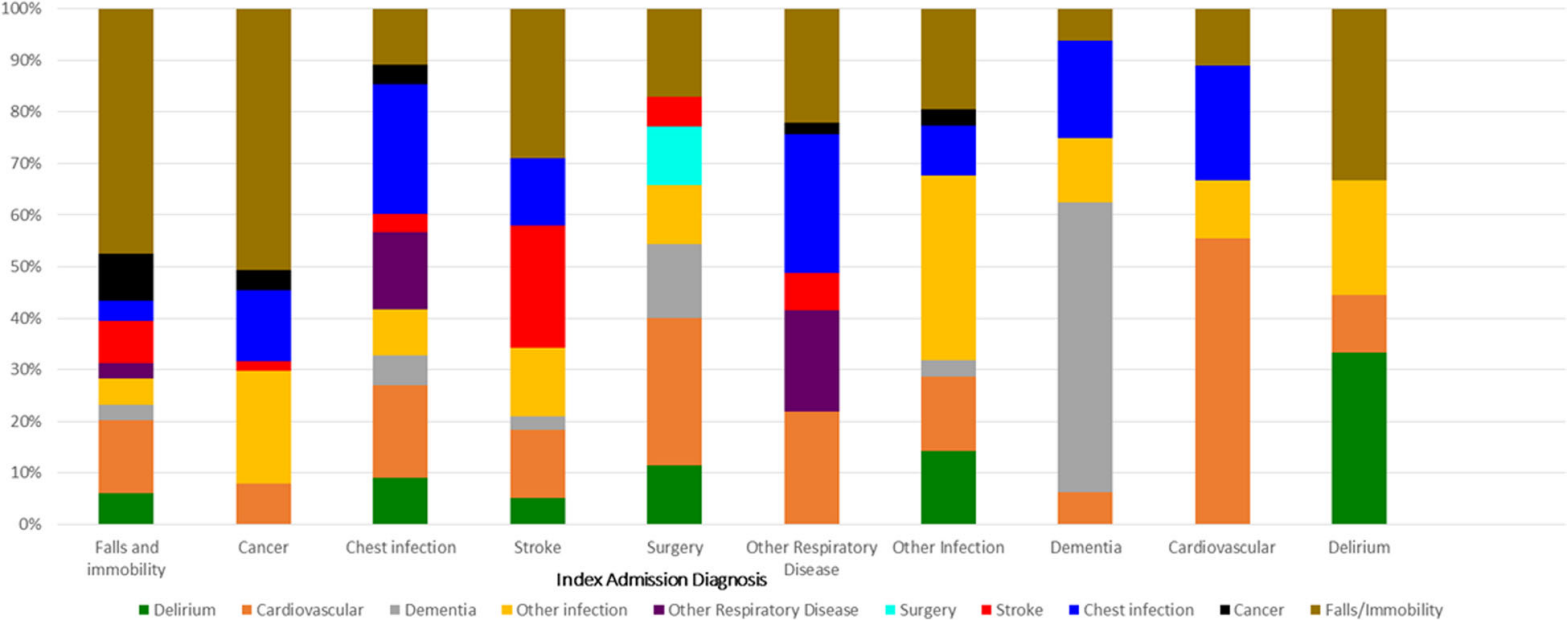
Diagnoses leading to readmission compared to first admission diagnoses (180 day readmissions)

Figures 1 and 2 show how both deaths and readmissions for the 30 and 180 day time periods varied over the study period. Figures 3 and 4 show the main readmission diagnoses at 30 days and 180 days respectively, broken down by the original admission diagnosis.

#### Multivariate analyses for acute hospital readmissions

Table 2 shows the results of univariate analysis for readmissions within 30 days and 180 days. Tables 3 and 4 show the results of multivariate regression analyses, conducted firstly using time to readmission as the dependent variable and censoring at death or end of the follow up period, and secondly using time to either death or readmission (whichever came first) as the dependent variable. Multivariate analysis showed that for patients readmitted within 30 days, older age, male sex, shorter length of hospital stay, discharge to own home and a previous diagnosis of chronic heart failure independent predictors of readmission within 30 days. For patients readmitted within 180 days, older age, male sex, shorter length of hospital stay and previous diagnosis of chronic heart failure were again independent predictors, but in addition previous myocardial infarction, previous diagnosis of cancer, and previous diagnosis of chronic obstructive pulmonary disease were additional independent predictors of readmission within 180 days. Results were very similar for readmission alone and for readmission or death as the outcome variable.

**Table 2:** Univariate Cox regression analyses – time to readmission

Variable in Analysis	Censored at 30 days		Censored at 180 days	
	Hazard Ratio (95% CI)	p	Hazard Ratio (95% CI)	p
Age (per year)	0.97 [0.96–0.99]	0.004	0.99 [0.98–0.99]	< 0.001
Female Sex	0.64 [0.49–0.83]	0.001	0.85 [0.80–0.90]	< 0.001
Admission Barthel score (per point)	1.05 [1.01–1.08]	0.01	1.01 [1.00–1.03]	0.13
Discharge Barthel score (per point)	1.01 [0.98–1.04]	0.34	1.01 [0.99–1.02]	0.39
Discharge Home	0.87 [0.65–1.16]	0.30	0.74 [0.64–0.85]	< 0.001
Length of Hospital stay (days)	0.997 [0.994–1.000]	0.04	0.998 [0.997–0.999]	< 0.001
Previous Myocardial Infarction	1.49 [1.09–2.03]	0.01	1.35 [1.17–1.56]	< 0.001
Previous Stroke	1.02 [0.61–1.69]	0.94	1.03 [0.82–1.29]	0.81
Congestive Cardiac Failure	1.90 [1.32–2.76]	0.001	1.68 [1.41–2.00]	< 0.001
Previous Diagnosis of Cancer	1.38 [0.96–1.99]	0.09	1.30 [1.10–1.53]	0.002
Diabetes Mellitus	1.11 [0.99–1.27]	0.09	1.04 [0.98–1.11]	0.19
Chronic Obstructive Pulmonary Disease	1.65 [1.19–2.28]	0.003	1.44 [1.24–1.68]	< 0.001
Medication Count on Discharge (per drug)	1.01 [0.98–1.05]	0.63	1.02 [1.00–1.03]	0.08
Maximum CRP Reading (per mg/L)	1.000 [0.998–1.001]	0.77	1.000 [0.999–1.001]	0.84
Time to half maximum CRP (per week)	0.997 [0.991–1.002]	0.23	1.000 [0.999–1.001]	0.87

**Table 3:** Multivariate Cox regression analysis – time to readmission censored at 30 days

Variable in Analysis	Risk of readmission (censored at 30 days or death)		Risk of readmission or death (censored at 30 days)	
	Hazard Ratio (95% CI)	p	Hazard Ratio (95% CI)	p
Age (per year)	0.98 [0.96–1.00]	0.04	0.98 [0.96–1.00]	0.06
Female Sex	0.76 [0.57–1.00]	0.05	0.76 [0.57–1.00]	0.05
Admission Barthel score (per point)	1.04 [1.00–1.08]	0.07	1.03 [1.00–1.07]	0.08
Discharge Home	0.54 [0.38–0.77]	0.001	0.51 [0.36–0.72]	< 0.001
Length of Hospital Stay (per day)	0.994 [0.991–0.998]	0.003	0.994 [0.990–0.998]	0.001
Previous Myocardial Infarction	1.25 [0.88–1.77]	0.21	1.19 [0.84–1.68]	0.32
Congestive Cardiac Failure	1.54 [1.02–2.34]	0.04	1.65 [1.10–2.47]	0.02
Previous Diagnosis of Cancer	1.33 [0.91–1.95]	0.14	1.30 [0.89–1.90]	0.18
Diabetes Mellitus	1.24 [0.89–1.72]	0.21	1.24 [0.89–1.72]	0.20
COPD	1.34 [0.94–1.90]	0.11	1.34 [0.95–1.90]	0.10

*COPD* Chronic obstructive pulmonary disease

**Table 4:** Multivariate Cox regression analysis – time to readmission, censored at 180 days

Variable in Analysis	Risk of readmission (censored at 180 days or death)		Risk of readmission or death (censored at 180 days)	
	Hazard Ratio (95% CI)	p	Hazard Ratio (95% CI)	p
Age (per year)	0.99 [0.98–1.00]	0.03	0.99 [0.98–1.00]	0.01
Female Sex	0.80 [0.71–0.91]	0.001	0.77 [0.69–0.87]	< 0.001
Admission Barthel score (per point)	0.99 [0.98–1.01]	0.44	0.98 [0.79–0.92]	0.03
Discharge Home	1.02 [0.86–1.21]	0.85	0.79 [0.68–0.92]	0.003
Length of Hospital Stay (per day)	0.997 [0.995–0.998]	< 0.001	0.995 [0.994–0.997]	< 0.001
Previous Myocardial Infarction	1.21 [1.03–1.42]	0.02	1.25 [1.07–1.45]	0.004
Congestive Cardiac Failure	1.48 [1.22–1.79]	< 0.001	1.57 [1.31–1.88]	< 0.001
Previous Diagnosis of Cancer	1.30 [1.10–1.55]	0.003	1.48 [1.27–1.73]	< 0.001
Diabetes Mellitus	1.00 [0.94–1.07]	1.00	0.99 [0.86–1.15]	0.93
COPD	1.24 [1.06–1.46]	0.009	1.23 [1.05–1.43]	0.009
Medication Count on Discharge	1.01 [0.99–1.03]	0.28	0.99 [0.98–1.01]	0.41

*COPD* Chronic obstructive pulmonary disease

The ability of these sets of predictors to discriminate between those readmitted and those not readmitted was limited, with a c-statistic of 0.64 (95%CI 0.60 to 0.68) for readmission within 30 days, and 0.59 (95%CI 0.57 to 0.61) for readmission within 180 days.

## Discussion

There are several key findings from this study. Readmissions to acute care in this cohort were due to a wide range of diagnoses, and were due to a different diagnosis to the index admission in over three-quarters of cases. Patterns differed between early and late readmission, and some index diagnoses (e.g. dementia, delirium, cardiovascular disease) were associated with a much higher chance of readmission with the same problem. The 30-day acute care readmission rate of 5.6% following a period of in-patient rehabilitation was lower than readmission rates reported in studies from the USA that ranged between 5.8–18.8% [10, 11, 19].

Risk factor patterns for early vs late readmission differed - for patients readmitted within 30 days a diagnosis of heart failure was the single factor increasing the likelihood of readmission, with discharge to the patients own home, and longer length of stay associated with reduced risk of readmission. In contrast, for patients readmitted within 180 days, the burden of comorbid disease as shown by a range of diagnoses and number of medications was associated with readmission. Although a longer length of stay was weakly associated with reduced risk of readmission to acute facilities, discharge to one’s own home was not a protective factor. The discriminatory ability of a combination of the above factors for early or late readmission was only modest and is unlikely to be helpful in clinical practice, despite the inclusion of a measure of functional ability. Markers of inflammation and of biological resilience (maximum CRP and rate of CRP recovery) were not associated with the risk of readmission.

Our findings are consistent with previous work from the USA, where two-thirds of readmissions were for a different problem than the index admission [2]. The even higher rate of discrepant diagnoses seen in our analysis is likely to be due to the older age and increased comorbidity of our study population. A large number of comorbid diseases means more opportunity for a problem to arise in a different organ system. Furthermore, although we did not measure frailty in our study population, it is highly likely that frailty was prevalent as is the case in other groups of older inpatients. Analysis of trends in English hospitals reported that overall frailty burden, based on the coding of at least one frailty syndrome, has increased from 12 to 14% between 2005 and 2013 for older patients admitted electively or acutely [20]. Frailty denotes a loss of homeostatic reserve across multiple body systems. Thus a disturbance or illness in one system can easily precipitate failure of a different system, which would be consistent with our findings.

The risk factors for readmission that were significant in our cohort are similar to those seen in other studies. Cancer, COPD, ischaemic heart disease, heart failure and stroke have all been associated with high readmission rates [21–23], and our results are consistent with previous studies where multimorbidity and previous hospitalisations were risk factors for readmission [24–27]. A study from the USA looking at readmission following a period of in-patient rehabilitation reported that heart failure, infections, nutritional and metabolic disorders alongside digestive disorders were the most common reasons for readmission [10]. We did not find that these last two diagnoses were commonly associated with readmission to acute care in our cohort.

Previous work has also shown that men are more likely to be readmitted to hospital within 30 days of discharge, possibly due to reduced health-seeking behaviors [28]. Differences in health-seeking behaviors, the lower role placed by men on preventative care and overly optimistic self-perceived health status may explain the apparent contradiction between higher morbidity in females in older age but higher risk of readmission for men after discharge [29, 30].

In contrast to previous studies from acute hospitals [9, 26, 27, 31], older age was associated with a reduced risk of readmission in our analysis. This may reflect patient selection – very old, very frail patients may not be selected for rehabilitation but may be transferred directly to nursing care facilities rather than the rehabilitation unit, whereas similarly frail younger patients may be selected for rehabilitation. Another possible explanation is that the rehabilitation team might view very old patients as at higher risk than younger patients, and accordingly plan discharges in such a way to mitigate this risk.

An association between shorter length of stay and increased risk of readmission has previously been reported for older patients discharged from acute hospitals [32–34]. However, we found only a small effect of length of stay on readmission risk; perhaps because patients admitted for rehabilitation have a relatively long length of stay, allowing comprehensive discharge planning and recovery from acute illness. The incremental benefit from an even longer stay may thus be minimal.

The discriminant ability of the risk factors we measured to predict future readmission was poor – too poor to be of use in planning clinical services. A systematic review for risk prediction models for hospital readmission reported that most current readmission risk prediction models, whether designed for comparative or clinical purposes, perform poorly [8]. The review looked at 30 studies that assessed 26 unique models, and commented that few of these examined variables associated with overall health and function, illness severity, or social determinants of health. This lack may be particularly important for older patients where social determinants of health alongside broader markers of function are crucial in terms of planning both primary, secondary and social care services.

Reducing readmissions in this patient group will be challenging. A systematic review of both in-hospital (17 studies) and home-care (15 studies) interventions aimed at reducing readmissions for in older people (> 75 years old) found that most did not have any effect on readmission [34]. However, those interventions with home-care components were more likely to be successful [34]. There is current work in the United Kingdom bringing together health and social care, in part to try and start addressing these concerns. However, the proportion of readmissions that are deemed avoidable after standardized and reliable review is not high; recent research reports less than 20% of readmissions are avoidable [27]. Furthermore, although readmission and hospitalization are important markers for disease severity, prognosis and quality of life there are clearly limits to any single metric as a surrogate for standard of care.

Our results reinforce the need to take a multisystem, holistic approach to reducing readmissions. Whilst some success has been noted with disease-specific interventions, e.g. for patients with heart failure [35], it is unlikely that interventions targeting a single disease (e.g. heart failure) will be successful in reducing readmissions due to other disease diagnoses after an index admission. Indeed, a focus on a single disease risks generating unintended knock-on consequences – rigorous control of heart failure may increase the risk of readmission with dehydration or acute kidney injury for example. Although a measure of biological resilience (CRP recovery rate) did not provide a useful way of predicting readmission in this analysis, similar measures of frailty or resilience may still provide both a way of predicting readmission and provide a target for intervention to reduce readmissions. Furthermore, other studies looking at readmission from rehabilitation units have suggested that information on functional status measures that are easily monitored by health care providers may improve plans for smooth transition of care delivery and aid the reduction of risk for hospital readmission [11].

Our analysis has a number of strengths. We used detailed health and functional outcomes data on a large set of patients undergoing rehabilitation in a medicine for the elderly unit. Studies to date have not assessed readmission following in-patient rehabilitation in a general older rehabilitation population, and there are differences between this group of patients compared to older adults discharged directly from acute hospitals [10, 11]. As this study analyzed routinely collected data, the data represents real-world clinical information that enables greater generalizability of the results.

There are several limitations that deserve comment. Our data were examined retrospectively and were not collected with this study in mind. Data quality is usually imperfect in datasets of routinely collected clinical data, and not all patients had Barthel scores available for analysis. Although the range of discharge diagnoses that we could classify from discharge coding data was wide, such data depends on both accurate diagnosis and accurate recording of the discharge diagnoses for coding, which is not always the case in routine clinical care. Use of this source of diagnoses prevented us from including poorly-coded diagnoses such as dementia, and alternative sources (e.g. primary care records) were not available for linkage at the time of our analysis. The large number of reasons for the index hospital admissions precluded easy use of these reasons as a variable in the analyses of risk factors for readmission, but future work using larger datasets may be able to address this issue.

Patients who have been admitted to a rehabilitation unit have the ideal opportunity for discharge planning in a clinical environment geared towards optimizing hospital discharges. The results of our analysis may not necessarily be generalizable to other patients groups with shorter length of stay and less comprehensive discharge planning. Out of hospital care services have developed considerably since 2012 (the end of study period). Changes have included early community intervention services, Hospital @ Home teams and use of step-up intermediate care beds rather than admission to acute units. These changes have taken place in our locality after the end of the period studied in this analysis.

## Conclusion

Our results confirm and extend previous work that readmissions of older people after hospital admission are due to a wide range of causes, and are often not due to a recurrence of the index problem. Work is needed to develop intervention packages that address readmission risks common to a range of diseases and syndromes of ageing, with a focus both on optimizing physiology, but also supporting patients and carers. In parallel, further work is required to identify those at highest risk of readmission so that such intervention packages can be targeted appropriately.

## Abbreviations

CCF: Congestive cardiac failure
CKD: Chronic kidney disease
COPD: Chronic obstructive pulmonary disease
CRP: C – reactive protein
eGFR: estimated glomerular filtration rate
HIC: Health Informatics Centre
ICD: International Classification of Diseases
IQR: Inter-quartile range
MDRD: Modification of Diet in Renal Disease Study (MDRD) equation
NHS: National Health Service
NI: Not included
SCI-DC: Scottish Care Information Diabetes Collaborative
SD: Standard deviation
USA: United States of America

## References

[1] Donze J, Aujesky A, Williams D (2013). Potentially avoidable 30-day readmissions in medical patients. Derivation and validation of a predictive model. JAMA Intern Med.

[2] Jencks SF, Williams MV, Coleman EA (2009). Rehospitalisations among patients in the Medicare fee-for service program. N Engl J Med.

[3] Reducing Readmissions. NHS Innovation & Improvement. Available from: http://webarchive.nationalarchives.gov.uk/20121108093302/http://www.institute.nhs.uk/quality_and_service_improvement_tools/quality_and_service_improvement_tools/discharge_planning.html Accessed: 13th Oct 2017.

[4] Krumholz HM (2013). Post-hospital syndrome- an acquired, Transient Condition of Generalised Risk. N Engl J Med.

[5] van Walraven JA, Taljaard M (2011). Incidence of potentially avoidable urgent readmissions and their relation to all-cause urgent readmissions. CMAJ.

[6] Billings J, Blunt I, Steventon A, Georghiou LG, Bardsley M (2012). Development of a predictive model to identify inpatients at risk of re-admission within 30 days of discharge (PARR-30). BMJ Open.

[7] Cotter PE, Bhalla VK, Wallis SJ, Biram RW (2012). Predicting readmissions: poor performance of the LACE index in an older UK population. Age Ageing.

[8] Kansagara D, Englander H, Salanitro A (2011). Risk prediction models for readmission rates: a systematic review. JAMA.

[9] van Walraven DIA, Bell C (2010). Derivation and validation of an index to predict early death or unplanned readmission after discharge after discharge from hospital to the community. CMAJ.

[10] Ottenbacher KJ, Karmarkar A, Graham JE (2014). Thirty-day hospital readmission following discharge from post-acute rehabilitation in fee-for-service Medicare patients. JAMA.

[11] Fisher SR, Graham JE, Krishnan S, Ottenbacher KJ (2016). Predictors of 30-day readmission following inpatient rehabilitation for patients at high risk for hospital readmission. Phys Ther.

[12] Witham MD, Ramage L, Burns SL (2011). Trends in function and Postdischarge mortality in a medicine for the rehabilitation Centre over a 10-year period. Arch Phys Med Rehabil.

[13] Lynch JE, Henderson NR, Ramage L, McMurdo MET, Witham MD (2012). Association between statin medication and improved outcomes during inpatient rehabilitation in older people. Ageing and Ageing.

[14] Beveridge LD, Ramage L, McMurdo MET, George J, Witham MD (2013). Allopurinol use is associated with greater functional gains in older rehabilitation patients. Age Ageing.

[15] Scottish Index of Multiple Deprivation. SIMD Results 2012. Available from: http://simd.scotland.gov.uk/publication-2012/. Accessed 10 Oct 2017.

[16] Levey AS, Bosch JP, Lewis JB, Greene T, Rogers N, Roth D (1999). A more accurate method to estimate glomerular filtration rate from serum creatinine: a new prediction equation. Modification of diet in renal disease study group. Ann Intern Med.

[17] The ICD-10 Classification of Mental and Behavioural Disorders: Clinical descriptions and diagnostic guidelines. Geneva: World Health Organization, 1992.

[18] Barma M, Goodbrand JA, Donnan PT, McGilchrist MM, Frost H, McMurdo ME, Witham MD (2016). Slower decline in C-reactive protein after an inflammatory insult is associated with longer survival in older hospitalised patients. PLoS One.

[19] Dara LC, Ingber MJ, Carichner JBA (2018). Evaluating hospital readmission rates after discharge from inpatient rehabilitation. Arch Phys Med Rehabil.

[20] Soong J, Poots A, Scott S (2015). Quantifying the prevalence of frailty in English hospitals. BMJ Open.

[21] Saunders ND, Nichols SD, Antiporda MA, Johnson K (2015). Examination of unplanned 30-day readmissions to a comprehensive cancer hospital. J Oncol Pract.

[22] Royal College of Physicians. National COPD audit Programme. COPD: who cares when it matters most? – outcomes report 2014. National Supplementary Report 2017. London.

[23] Rao A, Barrow E, Vuik S, Darzi A, Aylin P (2016). Systematic review of hospital readmissions in stroke patients. Stroke Res Treat.

[24] Donze J, Lipsitz S, Bates DW, Schnipper JL (2013). Causes and patterns of readmissions in patients with common comorbidities: retrospective cohort study. BMJ.

[25] Kahlon S, Pederson J, Majumdar SR (2015). Association between frailty and 30-day outcomes after discharge from hospital. CMAJ.

[26] Marcantonio ER, McKean S, Goldfinger M (1999). Factors associated with unplanned hospital readmission among patients 65 years of age and older in a Medicare managed care plan. Am J Med.

[27] Walraven v, Bennet C, Jennings A (2011). Proportion of hospital readmissions deemed avoidable: a systematic review. CMAJ.

[28] Woz S, Mitchell S, Hesko C (2012). Gender as risk factor for 30 days post-discharge hospital utilisation: a secondary data analysis. BMJ Open.

[29] Courtenay WH (2000). Constructions of masculinity and their influence on men's well-being: a theory of gender and health. Soc Sci Med.

[30] Kirchengast S, Haslinger B (2008). Gender differences in health-related quality of life among healthy aged and old-aged Austrians: cross-sectional analysis. Gend Med.

[31] Silverstein MD, Huanying Q, Mercer SQ (2008). Risk factors for 30-day hospital readmission in patients ≥65 years of age. Proc (Bayl Univ Med Cent).

[32] Dobrzanska L, Newell R (2006). Readmissions: a primary care examination of reasons for readmission of older people and possible readmission risk factors. J Clin Nurs.

[33] Preyde M, Brassard K (2011). Evidence-based risk factors for adverse health outcomes in older patients after discharge home and assessment tools: a systematic review. J Evid Based Soc Work.

[34] Linertova R, Garcia-Perez L, Vazquez-Diaz JR (2011). Interventions to reduce hospital readmissions in the elderly: in-hospital or home care. A systematic review. J Eval Clin Pract.

[35] Desai AS, Stevenson LW (2012). Rehospitalization for heart failure – predict or prevent?. Circulation.

